# Supplementary material for: Are carbon emissions trading and green financial instruments synergistic? -Comprehensive quantitative research based on content analysis
Source: PLoS One. 2024 Mar 7;19(3):e0298601. doi: 10.1371/journal.pone.0298601 (PMC10919601; doi:10.1371/journal.pone.0298601)
Supplement: S1 Data — (ZIP) [file pone.0298601.s001.zip › Data/地级市Z_F工作报告.zip!/║┌┴·╜¡/║┌║╙╩╨/└┤╫╘╣1⁄2╓┌║┼í╛┬φ┐╦╩2╛▌═°í┐_2010.docx]

各位代表：

现在，我代表市人民政府向大会作工作报告，请审议，并请列席会议的同志提出意见。

一、2009年工作回顾

2009年是极不寻常的一年，我市经济社会发展经受了历史少见的重大考验。面对复杂多变的严峻形势，我们在省委、省政府和市委正确领导下，把“保增长、保民生、保稳定”作为贯彻落实科学发展观的最大实践，坚定信心，积极应对，破解难题，围绕市委提出的“七个产值超百亿元产业”，深入推进“十项绩效工程”和“五个一”活动，有效缓解了国际金融危机带来的冲击，主要指标逆势攀升，多项经济指标增速位于全省前列，经济社会呈现加快发展的态势，圆满完成了市四届人大三次会议确定的目标任务。全市生产总值224.9亿元，增长14%。城镇固定资产投资97.9亿元，增长80%，增速列全省第一。社会消费品零售总额46.9亿元，增长18.5%。全口径财政收入17.3亿元，增长31.1%。城镇居民人均可支配收入10058元，农村居民人均纯收入5610元，分别增长23%和15.8%。万元地区生产总值能耗下降5.9%。

——重点项目建设加快，年度投资大幅增长。安排重点项目51项，完成投资60.6亿元，增长72.6%，投资额和增速均创历史最好水平。大多宝山铜矿整体开发、逊克翠宏山铁多金属矿和东安岩金矿、爱辉争光岩金矿、合作区5000吨物理法太阳能级多晶硅、龙江化工14万吨聚乙烯醇、海澜热电、孙吴热电、五大连池温都水乡国际旅游接待中心等项目加快建设，风景区40万吨天然矿泉水、爱辉20万千瓦风能发电等项目开工。五大连池和嫩江风能发电、二道坎水库、逊克乌二水电站等大项目前期工作有新突破。公路建设“三年决战”开局良好，嫩黑公路、吉黑高速北安至黑河段、加黑公路多宝山至黑河段、京加公路嫩江段和绥北公路北安段等项目开工建设，机场路改扩建完工。建成农村公路1465公里，北安所有行政村全部实现通村公路硬化。

——工业总量日益壮大，园区经济初具规模。预计规模以上工业增加值16亿元，增长20.6%；地方规模以上工业利税4.9亿元，增长74.4%。关鸟河水泥厂、兴边煤矿、东兴煤矿等近年投产的26户企业纳入统计，对工业经济增长起到重要支撑作用。省级以上工业园区达到5个，启动区面积5.5平方公里，入区企业50余户。合作区被列为全省六大新材料产业基地，五秀山俄电加工区耗电7亿度，实现增加值8120万元，税金1895万元，已形成工业硅产能14.5万吨、碳化硼和多晶硅各1000吨。北安工业园区基础设施投入4500万元，进发汽车配件等15户企业入区，辟建了中小科技企业创业中心，6户企业入区孵化。爱辉工业园区基础设施投入4000万元，林板一体化等项目开工建设。嫩江工业园区基础设施投入近亿元，金鑫饲料加工等9户企业进驻。五大连池工业园区已有苏打水开发等6户企业进驻。“三新”产业取得进展，爱辉薄膜太阳能大棚发电项目已在国家立项，并新建实验棚2栋。生物质发电、生物质秸秆制浆、生物制药及维生素C等项目正在加快推进。

——“双增”工程深入实施，农村经济全面发展。预计农业增加值55.6亿元，增长7%。粮食总产218.4万吨，增长8%。嫩江荣获全国产粮先进县称号。全市土地流转370万亩，适度规模经营610万亩。转移农村劳动力17.5万人，实现收入10.5亿元，分别增长2%和16%。畜牧业增加值7.3亿元，增长10%。奶牛、肉牛、大鹅产业带建设步伐加快，存栏分别增长10%、12%和10%。龙头企业实现销售收入27亿元，五大连池华滨小麦加工项目投产，嫩江嵩天加工城、北安汉麻纺织等项目开工，嫩江翔河油脂项目启动。林业产业产值15亿元，增长25%。按照三年造林规划，完成造林面积22.6万亩，占计划的108%。国有林流转52.5万亩，集体林流转开始试点。逊克北药种植规模不断扩大。北安、嫩江、爱辉节水灌溉工程全面完成，北安海星、逊克前进、爱辉卧牛湖水库除险加固工程主体完工。新建农村饮水安全工程90处，治理水土流失9.2万亩。新组建农机合作社17个，北安争取到全省两个现代农业整县推进试点之一。秋整地887万亩，其中深松418万亩。境外土地开发80万亩。争取国家大豆政策性收购计划118万吨，缓解了农民余粮销售难题。加强粮食仓储设施建设，新增标准仓容4.8万吨。建立淡季化肥储备制度，为农民减少投入1.9亿元。农民专业合作经济组织463个，带动农户6.8万户。场县共建取得新进展，农机跨区作业325万亩。启动城乡一体化试点乡镇20个、新农村建设第二批试点村59个。各县（市）区均被列入大小兴安岭林区生态保护与经济转型规划，有望得到国家更多资金扶持。五大连池被国家确定为资源枯竭型城市，已获得扶持资金1.3亿元。

——经贸合作迎难拓展，招商引资实现突破。受金融危机和政策调整等诸多因素影响，对外贸易进出口总值18.9亿美元，下降34.9%，降幅与全省平均水平基本持平。引进进口企业2家，具有经营权的外贸企业达到610家。以适应性战略调整为主导，坚持“走出去”，实施合作项目12项，合同金额1977万美元，完成森林采伐5万立方米、建筑面积12万平方米。大自然和东方明珠公司在俄煤炭采掘、鑫诚和龙洋公司森林采伐加工等项目开工，布市重汽4S店试营业。启动了合作区区域置换和跨境经济合作区可行性课题研究工作。黑龙江大桥和浮箱固冰通道项目取得进展。黑河港改造工程启动，口岸改造项目二期立项。组织了庆祝中俄建交60周年和中国“俄语年” 活动，开展了首次界河应急联合演习。黑河与布市建立了友好城市。创新招商引资方式，在市直16个重点部门分别抽调一名副职和两名工作人员，离岗招商。全市共落实项目237项，其中亿元以上22 项；到位资金55.3亿元，增长31.8%。引进外资企业6家，利用外资4355万美元。

——“四型经济”不断壮大，消费市场运行活跃。接待旅游者216万人次，增长26.5%。率先在中俄边境启动了旅游异地办证试点。编制了黄金旅游带、生态旅游以及黑龙江岛屿开发、瑷珲仿古街等旅游规划。开通了黑河—上海—三亚航线。五大连池风景区被命名为“中国矿泉城”，总体规划通过国务院批复，申遗文本通过世界遗产中心预审，火山矿泉文化魅力名镇建设启动，机场项目完成选址，北安至五大连池铁路项目预可研通过审定，火山博物馆正在规划设计。《闯关东》影视基地、卧牛湖、二龙山景区晋升国家3A级景区。五大连池被评为中国品牌旅游城市。山口自然保护区被命名为省级地质公园。中俄民族风情园俄罗斯风情街建成，孙吴胜山要塞景区道路完工，黑龙江知青博物馆开馆。开通了夜游黑龙江项目。试车企业和研发机构达到60家，成为中国汽协试验场分会成员。成功举办了大黑河岛国际经贸洽谈会、华侨华人黑河行、国际旅游与文化产业高峰论坛及边境地区中俄合作市长论坛等重大活动，组织和承办各级各类会议99次，聚集人员4万余人次。互贸区消费环境专项整治取得成效。新评定四星级和三星级酒店各2家。市区批发业、零售业、住宿餐饮业零售额分别增长20.4%、12.8%和22.3%。发放汽车和家电下乡补贴资金1153万元。推进“万村千乡市场工程”建设，农家连锁店和配送中心达到709家。农村消费品零售额10.3亿元，增长28.7%。

——财政收入再创新高，金融投入力度加大。全市财政一般预算收入9.9亿元，增长37.4%，增速列全省第一；一般预算支出57.7亿元，增长20.4%。市直全口径财政收入4.8亿元，一般预算收入2.3亿元，分别增长26.5%和28.5%；一般预算支出10.2亿元，增长29.4%。39个财源项目列入省重点跟踪推进项目。强化重点行业税收征管，煤炭税收2.1亿元，增长75%；房地产业税收7500万元，增长151.8%；建筑业税收1.8亿元，增长81.5%。规范政府收费管理意见相继出台，市直非税收入2.4亿元，增长23%。向上争取44.3亿元，增长10%。国库集中支付转轨改革全面完成，逊克在全省率先实现村级资金集中支付。市本级预算单位公务卡改革工作正在进行。大项目前期、公益事业和民生工程投入不断增加。政策性担保规模继续扩大，中小企业委托贷款和担保贷款1.4亿元。金融机构各项存款余额335亿元，各项贷款余额140亿元，分别比年初增长21%和46%。

——城乡建设统筹推进，城市品位有所提升。市区发展定位和风貌规划完成，总体规划修编启动，滨水区开发利用总体规划编制初步完成。北安、嫩江风貌规划完成初稿。全市城建基础设施投入6.6亿元，其中市区2.6亿元，龙海路和市区西部三条主要街路新建、通江路沥青罩面、环城南路人行道改造、铁路街拓宽以及绿化、亮化、小区庭院改造等工程竣工。污水处理厂和生活垃圾处理场投入运营，医疗废物处理厂完工。兴安街人防工程完成顶板建设和路面恢复，大黑河岛桥增幅加宽和步行栈道桥工程开工，城市雕塑和其他环境小品完成设计。新增城市绿化面积45.3万平方米，实施了“万棵果树进社区”活动，栽植花楸等果树6032株。城市“五化”管理全面启动，市区环卫作业、道路维修、亮化管护等全面实施市场化机制。各县（市）城市建设加快，嫩江环岛景观、北安铁西广场、逊克口岸路、孙吴和五大连池公园等工程均已完工。全市新开工建筑面积183万平方米，其中市区70万平方米。改造棚户区43.6万平方米，改造农村泥草房8802户，住房砖瓦化率68.8%；城镇和农村人均住房建筑面积分别达到25.2和19.4平方米。生态市建设进一步加强，在全省率先完成松花江流域污染治理项目，黑河热电、金星公司脱硫设施投入使用。

——社会事业全面进步，公共服务更加健全。获准国家和省级科技项目31项，其中2项被列入国家级中小企业创新基金项目，1项被列入省级重大科技攻关项目。招聘重点院校毕业生143名，其中研究生以上学历38名。小学和初中辍学率分别控制在0.01%和0.41%，高中阶段毛入学率65%。13所义务教育学校达到省级标准。新建和扩建3所特殊教育学校，实现特殊教育全覆盖。市青少年活动中心被教育部命名为中俄中小学生交流基地。市第一医院门诊楼、北安第一医院病房楼投入使用，市中医院搬迁新建项目开工，新建和改造16个乡镇卫生院。新型农村合作医疗农民参合率96.4%。新建和改扩建乡镇计生服务站19个，兑现了城镇无业居民和下岗职工独生子女父母奖励费，人口自然增长率2.8‰。市广播电视中心和发射塔完工，有线电视数字化整体转换工作顺利推进，新建21个乡镇综合文化站和75个农家书屋。承办了中宣部等十部委组织的“激情广场大家唱”、全省第四届少数民族文艺会演等活动。爱辉区被国务院授予全国民族团结进步先进集体。成功举办了全市第四届运动会，获得省级以上体育比赛奖牌99枚，青少年冰上活动中心完工。黑河地震台网中心完成整体搬迁和软硬件升级。妇女儿童、侨务、残疾人、档案、地方史志等工作都取得了新成绩。

——改善民生成效显著，安全稳定形势趋好。新增城镇就业1.97万人，完成省指标的152%，登记失业率3.6%，零就业家庭实现至少一人稳定就业。新建见习基地27个，安排见习毕业生1070人，毕业生就业率76.3%。发放小额担保贷款1.13亿元，扶持3615名下岗失业人员自主创业。社会保险覆盖面不断扩大，基本医疗、失业、工伤和生育保险参保人数均超额完成计划。城乡救助体系更加完善，实施了城乡一体化大病医疗救助，出台了城市低收入家庭认定管理办法，城乡低保月人均补差分别达到162元和68元。城乡优抚对象补助水平提高。启动实施了市区城镇居民基本医疗保险，市直住院医疗费最高支付限额由2万元提高到3万元，将部分门诊大病医疗费纳入统筹基金支付范围。为城镇低保无房家庭解决实物配租房978套，为18154户低收入住房困难家庭发放租赁补贴3263万元。新建和改造46个城市社区用房、20个农村样板社区服务中心。出台了老年人及老年服务机构优待意见，市老年公寓投入使用，北安、爱辉被列为全省居家养老示范县（市）区。信访工作不断加强，整合资源筹建了群众诉求调处中心，信访总量、人次分别下降46.9%和28.4%，复查复核息访率达到97%，并在国家层面介绍了经验。安全生产形势稳定，各类事故起数、死亡人数分别下降33.2 %和23.6%。探索建立森林防火“两个机制”和“五个系统”，连续七个防期未发生重特大森林火灾。市区出租车实现了统一标识，计价收费。开展了“文明过马路、礼让斑马线”活动，交通秩序明显好转。继续实施“警灯闪烁”工程，治安防范视频监控系统建设完成，刑事案件破案率73.2%，命案破案率95.5%，“平安黑河”建设成效显著，社会治安形势持续稳定。边境管理进一步加强，边境秩序安全稳定。

——自身建设不断加强，政府效能明显提高。主动接受人大依法监督和政协民主监督，163件建议和提案全部办结。严格实行行政执法责任制和执法过错追究制，依法行政工作扎实推进。拓展政务公开范围，加强电子政务建设，设置了政务公开触摸屏便民查询系统，36个单位便民服务指南在网上公布。加强应急管理机构建设，应急处置能力得到提高。全面实施“十项绩效工程”，开展“五个一”活动，完善激励奖惩机制。建立健全重大决策合法性审查制度，完善了行政问责体系。承接了国务院相对集中行政复议试点工作。开展集中整顿机关作风活动，工作效率明显提高。出台了进一步加强重点领域行政权力阳光操作意见，党风廉政建设进一步加强。

成绩来之不易，经验弥足珍贵。一年来的工作实践，使我们深刻认识到：必须把科学发展作为政府工作的第一要务。正确处理好应对当前危机与促进长远发展的关系，把学习实践科学发展观活动成果切实体现在科学审视市情上，体现在完善发展思路上，体现在一件事一件事抓落实上。坚决打好扩内需、保增长这场硬仗，在提质中求速度，在竞争中争位置，在发展中惠民生。必须把破解矛盾作为能力建设的根本要求。在严峻形势下，对矛盾不躲不闪，主动攻坚克难，无论是在面对企业发展、外贸下滑、支出增多等困难上，还是在应对甲型H1N1流感、生猪疫情等突发事件上，都主动上手，不断提高驾驭复杂局面的能力，在化解矛盾中破浪前行。必须把机制创新作为跨越发展的动力源泉。不断解放思想，坚持用改革的办法解决发展中的困难和问题，积极推进农村土地流转、城市管理市场化运作、财税征管以票控税、国有和集体林权改革、招商引资专业队伍建设、信访复查复核工作机制和政府运行模式创新，通过工作创新，重点难点问题的解决有了重大突破。必须把作风建设作为应对危机的有力保障。面对繁重的工作任务，根据不断变化的形势，充分调动各县（市）区和各部门积极性，研究解决影响工作的具体问题，主动跑省进京向上争取，深入基层调查研究，掌握上情，了解下情，求真务实，努力抓好各项工作落实。必须把改善民生作为政府工作的最终落脚点。时刻把群众的安危冷暖抓在手上，落实在行动上，出台了问需于民关注民生工作经常化意见，开展了关爱黑河最佳建议和最佳创意奖评选活动，建立了民情恳谈会制度，拓宽了群众反映问题、建言献策渠道。我们相信，在今后的前进道路上，无论有多少艰难险阻，无论有多少曲折困难，只要坚持和发扬这种团结一心、昂扬向上、顽强拼搏的精神，脚踏实地，埋头苦干，就一定能够把黑河建设得更加美好！

各位代表，过去的一年，我们之所以能够战胜各种困难，取得这些成绩，得益于省委、省政府和市委的正确领导，得益于人大、政协的全力支持，得益于全市人民的团结奋斗。在此，我代表市政府，向辛勤工作在各行各业的全市人民，向给予政府工作大力支持的各级人大代表、政协委员和各民主党派、工商联、无党派人士、人民团体，向关心支持地方工作的驻黑中省直单位和人民解放军、武警官兵，表示崇高敬意和衷心感谢！

在肯定成绩的同时，我们也清醒地看到，全市经济社会发展中还存在一些困难和问题。经济回升的基础还不牢固，保持又好又快发展难度仍然很大。农业基础设施落后，产业化水平低，粮食稳定增产和农民持续增收压力较大；工业经济规模小，产业链条短，牵动力强的大项目和高科技项目少；外经贸形势严峻，对外贸易进出口总值下降；财政收支矛盾依然突出，一些涉及群众切身利益的问题解决得还不够好。政府少数工作人员事业心不强、工作落实不力、办事效率不高，等等。对此，我们一定高度重视，采取有效措施加以解决。

二、2010年工作任务

2010年是“十一五”规划的最后一年，做好今年工作，对保持经济又好又快发展、启动实施“十二五”规划意义重大。我们要巩固应对国际金融危机取得的成果，紧紧抓住国家继续实施积极财政政策和适度宽松货币政策机遇，跟踪好国家宏观调控的重点、力度和节奏，努力做到外延扩展与内涵提升并重，突出扩展外延；加快发展与转变方式并重，突出在发展中转变；大项目和小项目并重，突出上大项目；质量与效益并重，突出在提高质量中增效益；“好”与“快”并重，突出在加快发展中求好。政府工作要全面贯彻党的十七大、十七届三中、四中全会及中央、省委经济工作会议精神，深入落实科学发展观，围绕省委提出的“八大经济区”和“十大工程”，进一步深化改革开放，抢抓政策机遇，扩大投资规模，壮大“七个产值超百亿元产业”，加快“五个行动区”建设，推进“三新”产业，继续实施“双增”工程，扩大消费需求，着力改善民生，维护安全稳定，以“十项绩效工程”为载体，推进经济社会更大更好更快发展。主要预期目标：全市生产总值增长11%。城镇固定资产投资增长35%。社会消费品零售总额增长15%。对外贸易进出口总值增长15%。全口径财政收入增长12%。城镇居民人均可支配收入增长8%，农村居民人均纯收入增长6%。万元地区生产总值能耗下降4.5%。着重抓好八项工作：

（一）强力推进大项目建设，发挥投资拉动作用，保持投资持续增长

突出抓好重大产业项目。开工和建成投产重点项目55项，年度计划投资140亿元。加快大多宝山铜矿、翠宏山铁多金属矿、东安岩金矿、14万吨聚乙烯醇及30万吨电石化工、1000万锭汉麻纺织加工等项目建设，力争年产30万吨欧林煤矿、争光岩金矿、库南铅锌矿、40万吨天然矿泉水、10万立方米林板一体化、庆华机械装备制造加工等项目投产。年产30万吨金三角煤矿进行全面改造。争取8万吨聚乙烯管材、100万吨生物质秸秆制浆等项目开工。

积极推进交通水电等基础设施项目。做好省管项目服务工作，加快市管和县管项目建设，确保完成公路建设“三年决战”年度计划。黑嘉公路竣工通车，京加公路讷河至嫩江段、吉黑高速北安至黑河段、绥北公路北安段、加黑公路卧都河至黑河段完成部分路面工程，嫩黑公路、前嫩公路北安至嫩江段、吉黑高速辅道开工建设，黑河外环路争取完工。新建农村公路600公里，年内全部竣工通车。争取1万千瓦乌二水电站开工建设。

高标准谋划储备一批重大项目。围绕“十二五”规划编制，精心谋划一批大项目，调整充实项目库，全市储备亿元以上项目28个。做实做细前期要件，组成专门班子，重点推进国电集团煤电一体化、二道坎水库、嫩江和五大连池风能发电、山口水库跨流域供水、秸秆密度板等项目前期。同时，进一步优化服务，搭建好政策、要素和服务平台，严格执行项目审批限时办结制，实行重点项目审批办结周报制。

（二）调整优化工业结构，扩大产业规模，提高工业经济整体素质

发展壮大“三新”产业。在继续扩张工业总量的同时，加快培育新兴产业，规模以上工业增加值增长20%，地方规模以上工业利税增长12%。扩大多晶硅产能，确保合作区5000吨物理法太阳能级多晶硅一期工程投产，积极发展硅晶切片、太阳能电池等光伏产业。推进爱辉20万千瓦风能发电项目建设，五大连池3万千瓦生物质发电、爱辉10兆瓦薄膜太阳能大棚发电项目开工。支持黑河三精药业、北安飞龙制药扩大规模，加快嫩江北药加工项目建设，开工合作区1万吨维生素C项目，完成爱辉6000万支HPV疫苗项目审批。经过一两年努力，各县（市）区至少有一个“三新”产业基本培育成型。

加快工业园区建设。推进合作区区域置换工作，加快五秀山和二公河园区基础设施建设，做到统一规划，协调发展。二公河俄电加工区建设220千伏变电所、7公里铁路专用线及路网。园区用电量达到10亿度。北安工业园区加快道路、供热等设施建设，引进更多企业进入创业中心孵化。嫩江工业园区完成规划和基础设施建设工作。其他县（市）区结合本地实际，认真做好园区规划，合理调整产业布局，进一步完善基础设施，引导生产要素向园区集中，各县（市）区力争园区企业数量都有一定增长。

在开发中延伸资源加工产业链。抓好矿产资源勘探开发总体规划修编，加大铜、铁等矿产资源勘探力度，加强对探矿权的监管，建立矿业权交易信息平台。对嫩江、逊克、爱辉已探明储量的矿产地，积极协调对接，办理审批核准手续，加快开发步伐。注重依托资源但又不完全依赖资源，立足现有的铜、铁、金、煤等资源条件，大力引进高科技项目，扶持企业上新工艺、新技术、新设备，加快下游产品开发，提高附加值，实现资源利用最大化。

抓好节能减排工作。大力发展低碳经济和绿色经济，转变发展方式。推进电力、冶金、煤炭、建材等重点行业节能降耗，搞好厂区园林化、公园化建设，培育环保型、清洁型产业。开展城市环境综合整治定量考核试验工作，建立重点监控企业污染物排放台帐，化学需氧量和二氧化硫排放量完成年度目标和“十一五”规划目标。完善黑河热电节能改造项目，推进北安污水处理厂、五大连池垃圾处理场建设。同时，加强工业经济运行监控，组织好煤电油运的供需衔接，出台中小企业扶持政策，切实解决影响企业生产经营的困难和问题。

（三）发展现代农业，壮大县域经济，加快城乡一体化进程

发展优质高效农业。继续实施“双增”工程，农业增加值增长4%，粮食单产提高5%。引导农民调整种植业结构，调减大豆种植面积，种植小麦、马铃薯、玉米和高效经济作物，大豆面积比重控制在70%以下。扩大绿色农产品生产规模，种植面积800万亩。推进畜牧业跨越发展工程，畜牧业增加值增长10%。发展特色休闲渔业，建设逊克、孙吴、爱辉网箱养殖基地，扩大北安河蟹养殖面积。培育壮大林业经济，林业产业产值增长20%。推进大型商品粮生产基地建设和北安现代农业整县推进试点。

加强农业基础建设。投资1.36亿元，推进北安、嫩江、五大连池节水灌溉示范区项目，建设灌溉沟渠和排涝沟渠，农田灌溉面积增长5%。力争黑龙江护岸工程在国家立项，做好引嫩工程、堤防工程和大小黑河岛、黄河口岛开发等项目争取工作。完成秋整地1000万亩，其中深松500万亩以上。在俄土地开发90万亩。加大农业综合开发力度，改造中低产田25万亩，建设高标准农田2万亩，投资增长10%。强化科技培训和技术配套，建立县级标准化农技推广中心3个，抓好大豆密植等增产技术推广应用。完善人工影响天气工程体系，做好新一代天气雷达项目前期工作。投资1.54亿元，新建中储粮北安直属库及黑河龙丰等粮食仓储库。

加快县域经济发展。科学编制县域经济发展规划，培育各具特色的支柱产业，稳定农业基础，扩大工业总量，发展第三产业，完善基础设施和公共服务体系，各县（市）区在全省县级经济发展速度上要确保升位。深化场县及林乡、企乡合作共建，整合医疗、教育等资源，加强人才交流，实现区域联动发展。加快北安赵光、爱辉罕达汽等20个城乡一体化试点乡镇建设，增强小城镇承载功能。采取有针对性的措施，增加农民收入。推进城乡户籍、就业、社保等制度改革，逐步实现城乡基本公共服务均等化。

建设生态保障体系。全面推进大小兴安岭生态保护与经济转型规划编制与实施。加强防护林体系建设，努力完成三年造林绿化任务。完成集体林权主体改革。全面停止林木商业性采伐，严厉打击毁林毁草毁湿开荒，加快自然保护区建设步伐。加强中小河流治理，完成规划编制和初步设计，做好二公河、法别拉河、老莱河、乌裕尔河等治理项目前期工作。争取更多资金，综合整治矿区环境，推进采矿区复垦、土地整理和生态修复，筹建罕达汽矿山公园，争取国家生态功能区更多的扶持资金。

完善农业发展机制。加大农副产品龙头企业建设力度，引导企业与农户建立利益联结机制，推进北安大豆系列产品加工、嫩江嵩天加工城、逊克北药生产基地、爱辉奶牛养殖及乳制品加工等项目建设，规模以上龙头企业达到50家。完善市场机制、信息网络和服务平台，推进土地流转和规模经营，新增土地规模经营100万亩，转移农村劳动力18万人。抓好农机合作社管理和装备建设，争取新建大型现代合作社22个，完成更新改造55个，每个合作社作业能力达到4万亩，农业标准化作业水平达到80%以上。加强农村合作经济组织建设，新建专业合作社50个，全省“寒地黑土”示范社30个。

（四）推进沿边开放带建设，实施适应性战略调整，提升对外开放水平

调整贸易结构。坚持以贸易促合作，以合作促发展，全面落实中俄两国合作规划纲要和省对俄合作适应性战略调整意见，优化贸易结构和商品结构。针对国际国内市场需求，鼓励并引导大集团、大企业开展对俄贸易，以木材、煤炭、电力、化工原料、能源、原材料为主的进口贸易增长15%，扩大以服装纺织、建筑及装饰材料为主的出口贸易，增加水泥、果蔬、农副产品等地方产品出口。巩固扩大布市及阿州市场，加强与萨哈、伊尔库茨克、后贝加尔等地对接合作，拓展经贸区域。

深化对俄合作。实施贸易与投资互动，完善优惠政策，加大扶持力度，积极引进经贸主体，对俄贸易占对外贸易比重达到35%以上。引导鼓励企业“走出去”，加快合作项目建设，年产50万吨水泥粉磨站、煤炭采掘等项目启动，重汽4S店和建筑材料综合市场投入运营。扩大在俄森林采伐、工程劳务规模，建筑面积12万平方米，“金色年华”别墅区开工。完成阿州—黑河油品储运项目核准立项，推进境外木材加工园区项目前期。继续举办大黑河岛国际经贸洽谈会。发挥互贸区作用，扩大中俄商品流通。

完善贸易通道。会同俄方开工建设黑龙江浮箱固冰通道，年内建成。商请俄方做好黑龙江大桥前期工作。启动建设黑河口岸储运仓库。做好跨境经济合作区争取工作。加大对俄协调力度，改善口岸环境，提高通关效率，货运量增长10%。重点解决好流冰期通关不畅问题，确保不出现中外旅客滞留现象。

强化招商引资。成立招商工作委员会。围绕重点产业和重点项目，突出重点区域和重点企业，在发挥现有16个驻外招商组职能基础上，继续扩大招商部门范围，引资额增长20%。组建矿产资源勘探公司，探索以资源换项目、引项目新路子。加强与高等院校对接合作，引进新技术、新项目、新专利。

（五）培育新兴消费热点，促进城乡消费，增强对经济发展拉动作用

加快发展“四型经济”。举全市之力推进五大连池风景区申遗和环境综合整治工作，确保申遗成功。全面启动火山矿泉文化魅力名镇建设，聘请国外专家搞好规划设计，打造世界旅游名镇。完成机场、铁路项目前期，争取明年开工。继续推进火山博物馆、矿泉理疗中心、格球山综合开发等项目。加快瑷珲历史陈列馆改陈建设，启动中俄民族风情园续建工程，推进黑河大峡谷、瑷珲仿古街、孙吴战争遗迹、大沾河国家森林公园、五大连池低碳滨水新区建设。继续开发黑龙江游船项目。做好风景区5A级、瑷珲历史陈列馆4A级、孙吴胜山要塞3A级景区争取工作。争创中国旅游最佳目的地。接待国内外旅游者244万人次，实现收入35亿元，均增长13%。抓好试车经济，完善配套服务，增加试车效益。召开黑河寒区试车国际研讨会。积极组织和承办各级各类会议、大型会展活动和重大体育赛事，活动数量、聚集人员分别增长20%。继续在央视等主流媒体宣传促销，提高知名度和吸引力。

扩大城乡消费规模。发展城区经济、场县经济，落实国家和省促进消费政策，城乡消费规模比上年有所提高。开发特色公寓式住宅，通过高端策划和市场促销，力争国内及俄罗斯公民来黑河购房“候鸟式”居住增长10%。继续落实家电、汽车、摩托车下乡和农机购置等补贴政策。加快黑河公路货运枢纽、北安贸易城等项目建设。大力发展社区服务、中介咨询、服务外包等新兴产业。第三产业总量比上年有所增加。

创造优良消费环境。坚持政府主导，设立旅游基金，支持旅游项目建设和产业发展。建设高档商务酒店、星级宾馆、家庭公寓和度假村，增加商业网点和购物场所，完善旅游标识、娱乐休闲等配套设施。做好旅行社管理、导游员培训及服务行业等级评定工作，提高服务质量，投诉率低于上年。推进社区超市、“万村千乡市场工程”和“新网工程”，完善城乡流通网络。健全维权组织，清理非法导购人员，保护消费者合法权益。

（六）做好财政金融工作，提高资金使用效益，增强保障能力

大力培植财源。支持重点财源项目建设，可支配财力比上年提高15%。利用财税杠杆，扶持税收主体和骨干税源。强化税源监控，建立财税库网络监控平台，促进税源依法转为税收。加强税收征管，在继续强化煤炭、建筑、房地产和公路建设等税收征管基础上，重点加大商业服务和汽车运输业征收力度。坚持依法治税，严厉查处拖瞒逃漏税行为，挖掘增收潜力，提高税收占财政收入比重。规范非税收入征缴，强化人防结建费收缴。

优化支出结构。优先安排保运转、保民生、促发展的重点支出，保障重大事项、重点领域和重点建设资金需求。规范部门预算管理，严格控制预算追加。从严控制一般性支出，全面推行公务卡管理。提高市直公务员和事业单位人员工资性津补贴标准。除极特殊需求人才，机关和事业单位一律不得超编进人，严格控制人员经费支出。加快“金财工程”建设，开展财政“监督年”活动，加强政府采购管理。继续推行财政支出绩效评价试点，提高资金使用效益。

做好向上争取工作。完善部门信息通报制度，加强绩效考核，重点在基础设施建设、新型产业发展、水土流失治理、矿山生态恢复、棚户区改造、财税收入分成和专项补助等方面争取支持，向上争取额度增长10%以上。做好沟通衔接，把握政策导向，力争有更多项目列入国家和省扶持计划。

推动金融业与地方发展相融合。加强政银企合作，支持金融机构工作，引导金融机构加大对地方发展支持力度，创建政银企共赢发展平台。落实金融机构支持地方经济发展考核奖励办法，提高金融机构积极性，为企业投放贷款额度增长20%以上。推进农村金融体系建设，支持符合条件的农联社组建农村银行。

（七）完善规划管理，提升品位和档次，建设宜居生态滨水园林城市

抓好总体规划调整和控详规划编制。继续按照“东扩、西改、南调、北优”发展框架，加快市区总体规划修编工作，完成市区西部和东部1025公顷控详规划，提高控详规划质量和覆盖率。做好水的文章，完成滨水区开发利用总体规划，高起点搞好黑龙江沿江带、大黑河岛、月牙湖等滨水景观设计。抓好滨水的路、楼、绿、游及亮化的提档升级。聘请国外高层次专家，突出老城区节点设计和新城区景观设计，进一步完善城市风貌规划。完成土地利用总体规划、城市总体规划、经济发展规划的统筹工作，做到统一协调。各县（市）都要开展城市风貌规划工作，不断完善城镇体系规划及专项规划。

抓好城市风貌规划实施。突出宜居性、生态性、连续性和艺术性，建设好景观城市。构建便捷城市路网，拓宽环城西路，完成王肃街东延工程，建设通江路至开发路的两条连接路网，实施王肃街、电业街沥青罩面，完成外环路建设，更换王肃街和中央街东段人行道板。主次干道完好率达到98%。完成大黑河岛桥增幅加宽和步行栈道桥工程。兴安街人防工程投入使用。突出北欧现代风格，完成通江路、环城南路等主要街路建筑立面装饰。美化亮化城市环境，实施大黑河岛西部生态功能区环境建设工程，环岛人行道、艺术地坪等项目完工，亮化滩头树群和两座新桥。开工建设市区东南部文化公园，完成广电中心公园建设。在黑龙江公园和中央商业步行街建设雕塑。抓好机场路、龙科街、铁路街等重点区域绿化，继续实施“万棵果树进社区”活动。完成39个旧小区改造，楼房区全部消灭裸露地面，达到绿覆盖和硬覆盖。加快推进各县（市）主要街路建筑立面装饰、绿化亮化及公园建设等工程。抓好“三供两治”、“一棚一草”改造和重点集镇、中心村规划建设，棚户区改造37.7万平方米，泥草房改造完成省下达目标。严格执行招投标制度，加强监督管理，确保工程质量。

抓好城市“五化”管理。坚持市场化、规范化、精细化、人性化、群众化，完善工作意见和实施方案，形成长效机制。突出政府花钱买服务，巩固和扩大市场化成果，逐步实现市政公用行业政府监管和专业养护分离。加强物业管理，提高服务水平。启动国家卫生城创建工作。建设数字化城管，三年内实现管理对象、管理过程、监控手段数字化。加强城市环境综合整治，开展违章建筑、建筑垃圾、洗浴行业烟尘污染等专项治理活动，特别是下大力气整治和规范临街商户广告牌匾，环境卫生群众满意率达到90%以上。各县（市）的城市管理水平也要全面提档升级。

（八）注重社会建设，努力保障和改善民生，让广大群众共享发展成果

高度重视科教和人才工作。深入贯彻落实全民科学素质行动计划纲要，不断提高未成年人、农民及城镇劳动人口、领导干部和公务员科学文化素质。加强与省内外大专院校和科研院所合作，加快科研成果引进和转化。推进硅基新材料产业基地建设，申报国家级TRIZ理论引进应用推广基地。统筹城乡教育资源，坚持中小学校长持证上岗制度，继续开展好教师岗位培训和新任教师培训，教育质量有明显提高。加强中小学生品德教育。进一步推进义务教育学校标准化建设，10所学校达到标准。大力发展职业教育，加强与项目单位合作，开展“订单式”技能培训。以俄罗斯“汉语年”为契机，打造全国知名俄语教育品牌。推进中小学校舍安全工程建设。制定好“十二五”人才队伍建设规划，出台优惠政策，重点引进教育、卫生、城建等领域急需领军人才，加快引进产业带动型和创新型人才。重视培育本地人才，加快培养农村实用人才。

提高群众健康水平。积极稳妥地推进医药卫生体制改革，抓好基本医疗保障和基本药物制度建设，年底前60%的基层公办卫生机构实施基本药物制度，强化医德医风建设，解决群众反映强烈的看病难、看病贵问题。筹建市药品采购中心。加强社区卫生服务站建设，加大公益项目投入力度。继续加大甲型H1N1流感、肺结核等传染疾病防控力度，建立药品不良反应预警机制。完成8到15岁儿童乙肝疫苗补种，继续开展65岁以上老人免费体检工作。巩固新型农村合作医疗成果，报销封顶线达到当地农民人均纯收入6倍以上。市第一医院争创三级医院，市中医院主体工程完工。推进人口“惠家工程”，稳定低生育水平，人口自然增长率控制在4.8‰以内。

丰富群众文体生活。开展“城市之光”和“金色田野”活动，办好第二十三届“黑河之夏”艺术节，创作一批文艺精品。继续推进中俄文化艺术交流。加强非物质文化遗产保护，完成鄂伦春族摩苏昆国家级项目整理工作。市广播电视发射塔投入使用，完成全市数字化整体转换及913台和810台整体搬迁工作。贯彻落实国务院《全民健身条例》，加强公共体育设施建设。

保障群众安居乐业。支持非公有制企业发展，推动全民创业，自主创业率高于上年。继续开展“双创”活动，安排毕业生见习岗位1000个，开发公益岗位1000个。城镇新增就业1.3万人，登记失业率控制在4.5%以内。加大保障性住房供给，建设实物配租房，租赁补贴1.8万户。加快城乡社区建设，加大投入，完善设施，50%城市社区达到和谐社区标准，新建改造30个农村样板社区。完善社会救助体系，搞好城市低收入家庭认定，加强城乡居民最低生活保障管理。抓好乡镇公共服务中心建设。扩大城镇居民基本医疗保险覆盖范围，搞好城乡医保统筹，抓好农村医保试点。妥善解决低保家庭、残疾人等困难群体医疗保障。启动新型农村养老保险试点，落实完善失地农民养老保险政策。建设市流浪未成年人救助保护中心和儿童福利院。

维护社会安定和谐。全面落实安全生产责任制，加强对重点行业、领域安全隐患排查和专项整治，严防重特大事故发生，安全生产不突破省下达指标。加快森林防火“两个机制”和“五个系统”建设，确保不发生重特大森林火灾。强化信访工作，发挥好群众诉求调处中心和信访复查复核委员会作用，有效预防和妥善处置群体性事件，越级上访率低于上年。进一步加强社会治安综合治理，提升“四个能力”。继续实施“警灯闪烁”工程，降低发案量，提高破案率。深入开展“文明过马路、礼让斑马线”等活动，防控交通事故，提高市民文明素质。加强法制宣传教育，做好“五五”普法验收工作。加强边境管理，确保边境安定。认真开展第六次全国人口普查工作。加强国防教育，继续保持“双拥模范城”荣誉称号。进一步做好民族宗教、妇女儿童、防震减灾、侨务、残疾人、档案、地方史志等工作。

三、全面加强政府自身建设

坚持用科学发展观武装头脑，不断解放思想，努力开拓视野，增强驾驭复杂局面和经济工作的能力。切实加强制度建设，把心思放到发展上，把精力放在落实上，努力建设负责任、有作为、高效率政府。健全科学决策、民主决策的长效机制。认真贯彻落实国务院《依法行政实施纲要》和《关于加强市县依法行政决定》，抓好规范权力运行机制建设，严格依照法定权限和程序行使权力。健全重大决策合法性审查制度，使决策更加符合客观实际，更加符合群众利益。实行重大决策评估、公示和听证制度，自觉接受市委领导，主动向人大报告工作、向政协通报情况，及时办理人大代表议案、批评、意见和政协委员提案，加强与民主党派、工商联、无党派人士和群团组织沟通。营造权责统一、服务优良的发展环境。积极稳妥推进政府机构改革，转变职能，优化组合，强化社会管理和公共服务。实行机构编制总量管理，从严控制机构设立、升格和编制增长。进一步调整和减少行政审批事项，简化审批流程，提高办事效率。对重大投资项目实行领办代办和全程跟踪服务，严肃查处影响企业正常生产经营行为，企业投诉处理率100%。完善政府诚信机制，努力建设诚信政府。加强政府网站建设，增加政务公开触摸屏俄文查询内容，逐步扩大政府外网范围，在政府部门内试行无纸化公文传输。发扬心系群众、务实高效的扎实作风。继续实施“十项绩效工程”，将这次会议通过的工作任务和市委全会暨经济工作会议确定的工作任务分解落实，明确责任，加大推进力度。腾出更多精力深入基层调查研究，实现问需于民关注民生工作经常化、制度化。切实提高执行力，围绕中心工作量化目标任务，加大问责力度，对重点工作已经落实的问效果，正在落实的看进度，尚未落实的查原因，落实不力的追责任。健全突发公共事件应急机制，提高应对能力。树立勤俭办事、廉洁从政的良好形象。切实加强公务员队伍廉政建设，严格执行廉洁自律各项规定。牢记“两个务必”，坚持量力而行，勤俭办事，坚决反对和制止奢侈浪费。构建完善有效的廉政监督机制，健全建设工程招投标、经营性土地使用权出让、政府采购和产权交易等规范权力运行体系，用制度管权、按制度办事、靠制度管人，从源头上预防和治理腐败。

各位代表，在困难和挑战面前，唯有实干才有出路，唯有发展才有希望。让我们在省委、省政府和市委的坚强领导下，紧紧团结和依靠全市人民，谋发展之势，鼓实干之劲，聚和谐之力，为全面完成“十一五”规划目标任务，推动经济社会更大更好更快发展而努力奋斗！
